# Genome Sequence of *Mycoplasma hyorhinis* Strain DBS 1050

**DOI:** 10.1128/genomeA.00127-14

**Published:** 2014-03-06

**Authors:** Alena Dabrazhynetskaya, Valerii Soika, Dmitriy Volokhov, Vahan Simonyan, Vladimir Chizhikov

**Affiliations:** Center for Biologics Evaluation and Research, Food and Drug Administration, Rockville, Maryland, USA

## Abstract

*Mycoplasma hyorhinis* is known as one of the most prevalent contaminants of mammalian cell and tissue cultures worldwide. Here, we present the complete genome sequence of the fastidious *M. hyorhinis* strain DBS 1050.

## GENOME ANNOUNCEMENT

*Mycoplasma hyorhinis* strain DBS 1050 belongs to cultivar α strains, which cannot be cultivated on the conventional cell-free media normally used to grow mycoplasmas, including *M. hyorhinis* type strain BTS-7 (1). It was shown that noncultivable cultivar α strains are sensitive to some growth-inhibiting factors present in commonly employed bacteriologic media (2). However, they grow efficiently in various cell and tissue cultures (1–3).

Although about 60% of *M. hyorhinis* strains detected in mycoplasma-infected cell lines belong to cultivar α (1), no genome from this group has been sequenced yet. We present here the first complete genome sequence of the *M. hyorhinis* cultivar α strain DBS 1050 (clonal isolate 3T-6, ATCC 29052).

The sequencing of a shotgun library of *M. hyorhinis* strain DBS 1050 was carried out by Axeq Technologies (Rockville, MD) using the GS FLX Titanium 454 sequencing platform. The resulting 117,624 sequence reads provided 47× coverage of the mycoplasma genome. About 99.95% of the reads were assembled into a single DNA molecule using the Hexagon assembler at the HIVE portal (FDA, Rockville, MD). The resulting genome was autoannotated via the Institute for Genome Sciences (IGS) Annotation Engine (University of Maryland School of Medicine, Baltimore, MD). Each gene was manually curated using the Web-based Manatee annotation tool.

The complete genome of *M. hyorhinis* DBS 1050 consists of a circular chromosome of 837,447 bp, with an overall G+C content of 25.9%. The genome contains 782 genes, of which 737 are coding sequences (CDSs), 33 are RNA genes, and 12 are pseudogenes. Among 737 potential protein-coding genes, 64.1% encode proteins with assigned functional role categories, 15.1% encode proteins with domain or family assignments, 8.4% encode hypothetical proteins with no significant sequence similarity to the other proteins, and 12.2% encode conserved hypothetical proteins with similarities to the other hypothetical proteins. More than 180 genes encode integral proteins or contain functional transmembrane domains.

The genome contains a single copy of the 5S rRNA gene separated from the 16S-23S rRNA operon. Two copies of the full-length IS*1221* transposase gene, described previously (4), as well as 20 degenerate copies of this gene with multiple frameshift mutations, are mapped in the same locations as those in *M. hyorhinis* strain GDL-1 (5). The variable lipoprotein (*vlp*) locus of DBS 1050 is slightly different from those of the other *M. hyorhinis* strains (5–8) and is represented by six *vlp* genes divided by two degenerate IS elements: 5′*-vlpD*-*vlpE*-*vlpF*-IS-*vlpA*-IS-*vlpB*-*vlpC*-3′***.***

### Nucleotide sequence accession number.

The genome sequence of *M. hyorhinis* DBS 1050 has been deposited in GenBank under the accession no. CP006849.
